# Philadelphia Almshouse Infirmary

**Published:** 1829-07

**Authors:** 


### PHILADELPHIA ALMSHOUSE INFIRMARY.

### *Case of Fever, with Death from Mercurial Irritation.* By Dr. Samuel Jackson.

T. Kelly, aged twenty-two years, entered the clinical ward, September 26th, 1818; his habits of life regular; a weaver by trade. He had been ill seven weeks with fever, which in the commencement had been pronounced, by his attending physician, bilious fever.

The symptoms on admission were, emaciation; cough troublesome at night; sense of stricture in chest; soreness of epigastrium, with tenderness and pain on slight pressure; hiccup; skin warm, dry, and sallow; pulse frequent, some fulness; sordes on teeth; tongue clean, dry and polished; eyes sallow; carotids pulsate with force; perfectly sensible; great soreness of his flesh; no appetite, and not troubled with thirst; diarrhœa, bowels moved six times before admission today, and almost constantly since; discharge watery and offensive. He appeared much prostrated and exhausted by his removal to the Infirmary.

He stated that during his illness he had been vomited several times, had been very frequently purged, and had been taking powders of calomel.

Directions were given to sponge his body with tepid whiskey, to have cold applications maintained to his head, with small quantities of weak wine whey until he should recover from the effects of his fatigue, and chicken-water for diet. Sulph. Quinæ gr. i. in mucilag Gum Arab. was directed every three hours; and injections of Laudanum gtt. xx. with flaxseed mucilage, to check the diarrhœa.

In the evening, improved; skin moist, and of natural heat; pulse seventy-six; tongue moistened. Omitted sponging and cold to head. Chest relieved.

27th.—Skin of natural warmth and moisture; tongue slightly furred, tumid, not florid, but disposed to become dry; pulse ninety-six, soft; abdomen tumid; right hypochondrium soft, but painful when pressed; left is occupied by a large hard tumor, extending to the umbilicus, but does not possess much sensibility; epistaxis; sordes on teeth. Vomited in afternoon. Lime-water, with arrow-root and gum-water for diet. Bowels too open.

28th.—Skin warm; tongue dry, nearly clean; pulse 100 and irritated ; nausea. Omit Sulph. Quinæ; pediluvium, and sponge the body as before.

Evening.—Febrile irritation declined; disorder of bowels checked.

29th.—Slept well. Skin moist, cool, and comfortable; no fever; pulse seventy-two, soft; tongue with adhesive mucus on it; slight hiccup and occasional retching; eyes jaundiced; abdomen covered with numerous purple spots. Beef-tea for diet, alternated with oyster-liquor.

30th.—No pain; tongue same; skin warmer; pulse more irritated; bowels disposed to be too loose. Tepid sponging, and injection of flaxseed mucilage and laudanum.

October 1st.—No fever; appears to be much improved.

2d.—Pulse more irritated; tongue become drier and florid; sordes collecting on teeth; diarrhœa; abdomen tympanitic or meteorized. Stimulant frictions to the skin, and lower extremities to be enveloped in blankets wrung out of warm water; injections of flaxseed and laudanum; fomentations to abdomen.

3d.—Pulse excited; skin warm; tongue florid; parotid glands swelled and painful. Poultice, moistened with lead-water, to parotids.

4th.—Swelling extends over the whole jaws and face; skin stretched and shining. Mouth cannot be opened to admit of an examination. Skin hot; feet cold. Milk and oyster-soup for diet.

5th.—Swelling increasing. Directed leeches to parotids. Breath offensive, mercurialized. The mercurial action has developed violent inflammation of all the salivary glands and mucous membrane of mouth, and which has extended to the adjacent tissues. No secretion of saliva from excess of irritative action.

6th.—Swelling extends down the neck on each side to near the clavicle; skin very tense, but not discoloured, and the swelling is very tense, as if the fascia was put on the stretch; pressure is exceedingly painful. No fever.

7th.—Nearly in same state; cooling lotions applied to swelling; sleeps well. Soft egg added to diet.

8th.—Suppuration of tumor in the parotids advancing. Poultice.

9th.—Tumor opened, discharged large quantity of healthy pus. Relieved of pain.

10th and 11th.—Discharge of pus very copious.

12th.—Had chill; diarrhœa came on; pulse very feeble.—Carb. Ammon. julep ℥ss. every hour; oyster-soup; beef-tea.

13th.—Slept well; discharge from abscess very profuse; pulse extremely feeble; strength failing. Diffusible stimulants exhibited ineffectually, and he died in the evening.

Case reported by Dr. Ashmead.

The body was removed by the friends of the deceased, who would not permit an autopsy to be performed.

*Remarks.*—This case, when first admitted, presented the characters attending on the inflammations of the mucous tissue of the digestive canal, assuming a chronic state, and which so frequently are the concluding act of bilious and remitting, and sometimes of intermittent fevers, treated improperly by emetics, active cathartics, and other irritating remedies, addressed to a tissue already morbidly irritated.

When fevers have passed to this state, they generally are christened typhus; and, when the meningeal membranes or the brain partake of the same condition, this designation is pronounced without hesitation; and, if violent stimulation be the treatment adopted, typhus gravior, in its most characteristic shape, soon grows up under the hand of the practitioner.

On admission into the ward, the case seemed too desperate to permit an expectation of recovery to be entertained. An improvement did take place, and a fair prospect of restoration appeared to be opening, when an increase of irritation ensued, for which no cause could be at first assigned; swelling of the parotids and jaws soon followed, the origin of which, although not at first suspected, evidently arose from the action of the mercury, which had been administered previous to his entrance into the house, and had been taken during his protracted illness. No circumstance could have been more untoward than this accident. The recuperative powers of the economy were exhausted by the severe disease that had preceded, and were at best merely capable of rescuing him from the chronic inflammations that had been permitted to become established in the digestive mucous tissue; and which event was even a problematical occurrence. The development of the mercurial irritation in this state, and to the extent it assumed, was a reinforcement in favor of the disease, and to the detriment of the patient, that gave a decided turn to the contest against him.

This result of the attempt to cure fevers by the establishment of a salivation, or to place the economy under the comprehensive domination of the mercurial irritation, is, I have strong grounds to believe, a circumstance far more common than is suspected, or than many will be disposed to acknowledge.

This fashionable practice I have abandoned since the epidemic of 1822, the commencement of the series of epidemic intermittent, remittent, and bilious fevers, that continue still to prevail over so large a portion of the country. I was compelled to renounce it from witnessing, in so many instances, the injurious results of the treatment to the patient. The mercurial action in violent cases, I found, could very rarely be brought on before the intensity of the local inflammations and the sympathetic fever were on the decline; and then the inflammations awakened by the mercurial irritation were not to be desired: they were nearly as much to be dreaded as those which constituted the disease. In many cases, after convalescence had commenced, the mercurial action came on, and I had the mortification to be perfectly convinced, though no suspicion crossed the mind of the patient, that a rapid recovery had been prevented, and protracted suffering been endured, in consequence of the employment of the remedy, though done under the sanction of high authority at home and "great names abroad."

The mercurial irritation, it is to be kept in mind, when it is developed as febrile disturbances in the system are subsiding, does not, in numerous instances, induce a salivation; nor is this effect a necessary consequence of the administration of the mercurial preparations. On the contrary, they often excite, at that time, from the numerous irritations still existing, an extent of action in the mucous tissue of the digestive canal and the mouth, and which is thence extended into the glands connected with them, transcending the degree in the range of which secretion is possible. The consequence is inflammation, ulceration, hemorrhages, together with re-excitement of febrile commotion. The new train of symptoms are then frequently set down for a relapse; and, if the mercurial treatment be instituted, the patient almost assuredly perishes.

Another effect of the mercurial irritation suddenly displayed in a system exhausted by an attack of fever, is prostration of the powers of the nervous system and of the heart, with a rapid collapse terminating speedily in death. The patient, in these instances, appears to be entering into convalescence; the fever has ceased from two days to six or seven; the appetite is improving; the skin is moist, but has a flabby feeling; head unembarrassed; bowels regular. In the midst of these favorable appearances, while all apprehensions are allayed, and a restoration is regarded as beyond a doubt, the patient, in all the cases that have come to my knowledge, is seized in the night with nervous tremors, cold sweats, great anxiety, rapid sinking of the forces, and, after a short agony, (often before the physician, who has been summoned on the invasion of the danger, can reach his patient,) the mortal scene has closed. The cases in which the circumstances I have mentioned occurred, were those in which the system had been freely charged with mercury during the febrile period, but no affection of the gums had taken place.*

---

Dr. Jackson also relates a case of bilious fever, with death from mercurial irritation. In this instance the patient continued to improve until the mercurial action had commenced. " From that time the case assumed a new aspect: inflammation of the gums and parietes of the mouth, and of the salivary glands, came on; hemorrhage from the mucous membrane of the mouth, fever, incapacity of swallowing, and death; which could not be attributed to any other cause than the mercurial irritation and fever that had been established."

* American Journal of Medical Sciences.

